# Comparison of the Effect of Plasma-Activated Water and Artificially Prepared Plasma-Activated Water on Wheat Grain Properties

**DOI:** 10.3390/plants11111471

**Published:** 2022-05-30

**Authors:** Jana Jirešová, Vladimír Scholtz, Jaroslav Julák, Božena Šerá

**Affiliations:** 1 Department of Physics and Measurements, University of Chemistry and Technology, Prague, Technická 5, 166 28 Prague, Czech Republic; vladimir.scholtz@vscht.cz; 2 Institute of Immunology and Microbiology, First Faculty of Medicine, Charles University and General University Hospital in Prague, Studničkova 7, 128 00 Prague, Czech Republic; jaroslav.julak@lf1.cuni.cz; 3 Department of Environmental Ecology and Landscape Management, Faculty of Natural Sciences, Comenius University in Bratislava, Ilkovičova 6, 842 15 Bratislava, Slovakia; bozena.sera@uniba.sk

**Keywords:** nonthermal plasma, plasma-activated water, seed treatment

## Abstract

Recently, much attention has been paid to the use of low-temperature plasmas and plasma-activated water (PAW) in various areas of biological research. In addition to its use in medicine, especially for low-temperature disinfection and sterilization, a number of works using plasma in various fields of agriculture have already appeared. While direct plasma action involves the effects of many highly reactive species with short lifetimes, the use of PAW involves the action of only long-lived particles. A number of articles have shown that the main stable components of PAW are H_2_O_2_, O_3_, HNO_2_, and HNO_3_. If so, then it would be faster and much more practical to artificially prepare PAW by directly mixing these chemicals in a given ratio. In this article, we review the literature describing the composition and properties of PAW prepared by various methods. We also draw attention to an otherwise rather neglected fact, that there are no significant differences between the action of PAW and artificially prepared PAW. The effect of PAW on the properties of wheat grains (*Triticum aestivum* L.) was determined. PAW exposure increased germination, shoot length, and fresh and dry shoot weight. The root length and R/S length, i.e., the ratio between the underground (R) and aboveground (S) length of the wheat seedlings, slightly decreased, while the other parameters changed only irregularly or not at all. Grains artificially inoculated with *Escherichia coli* were significantly decontaminated after only one hour of exposure to PAW, while *Saccharomyces cerevisiae* decontamination required soaking for 24 h. The differences between the PAW prepared by plasma treatment and the PAW prepared by artificially mixing the active ingredients, i.e., nitric acid and hydrogen peroxide, proved to be inconsistent and statistically insignificant. Therefore, it may be sufficient for further research to focus only on the effects of artificial PAW.

## 1. Introduction

In recent years, there has been increased interest in the use of low-temperature plasma for various treatments of agricultural products and foods. The rich literature on this topic is summarized in several review articles, e.g., [1,2,3,4,5]. The inactivation of phytopathogenic bacterial and fungal cells by various plasma sources was reviewed in [6,7]. The principles of each procedure and their significance are discussed in more detail in the works cited below.

### 1.1. Effect of Plasma on Seeds

Many studies have been devoted to the effects of direct plasma application on germination and other properties of seeds, including wheat; the germination of *Penicillium* spores may be also affected [8]. References [9,10,11,12,13,14,15,16,17,18,19] are several recent works in this area.

The role of nonthermal plasma in the development of plants from seeds to crops, or so-called “plasma agriculture”, has been reviewed in detail by [20,21,22].

### 1.2. Plasma-Activated Water (PAW)

In addition to the direct action of plasma, the action of plasma-activated water (PAW), produced by the exposure of water or aqueous solutions to plasma, has also received considerable attention. In the early stages of the research into this substance, it was called plasmatic acid [23] or water of death [24]. PAW retains its biological activity for a long time, more than one year after the cessation of plasma exposure [25].

PAW has received significant attention from researchers over the last decade due to its nonthermal and nontoxic mode of action, which relies on the interaction between reactive species and microbial structural components, leading to the inactivation of microbes [26,27]. A number of reactive chemical species causes the effects of PAW, especially its decontamination capabilities. Their abundance and representation depend strongly on the experimental conditions of PAW preparation, including the arrangement of the production discharge and the composition of the working gas. Many authors, e.g., [28,29,30,31,32], have described the properties of PAW. The advances in the methods for preparing PAW and monitoring its properties, as well as some of its practical applications, were recently reviewed by [33,34,35]. The possibility of preparing “artificial” PAW by mixing its chemical components was proposed and discussed by [24,36].

The efficacy of PAW against common plant microbial pathogens has been demonstrated [37], and the possibilities for using PAW in agriculture are summarized in [38]. For potential biomedical use, it is significant that PAW shows only scarce toxicity in live animals [39].

### 1.3. Reactive Particles of PAW

Numerous reactive chemical species are responsible for the decontamination capabilities of PAW. Generally, these are oxygen and nitrogen compounds known as ROS (reactive oxygen species) and RNS (reactive nitrogen species). Their abundance and representation depend strongly on the experimental conditions of PAW preparation, including the arrangement of the production discharge and the composition of the working gas. With the exception of nitric acid (HNO_3_), which is responsible for the persistent acidity of PAW, and the moderately stable ozone (O_3_) and nitrous acid (HNO_2_), they are generally unstable particles with short lifetimes. For example, the lifetime of OH radicals was determined to be in the order of µs by [40].

In [41], the authors attributed the bactericidal activity to a short-lived chemical species and suggested that it is rapidly lost, with O_2_^−•^ being important for this effect. These results are congruent with the hypothesis that the bactericidal species in PAW is not peroxynitrous acid (ONOOH), but peroxynitric acid (O_2_NOOH). The mechanisms of PAW action were described in detail by [42]. The possible mechanisms of the reactions occurring at the plasma/water interface have also been studied in detail by Volkov et al. [43]. According to some studies [44], the presence of certain metal ions, especially copper and zinc, can also have a significant impact on the effectiveness of activated water. Medvecká et al. [45] described the dependence of the composition of reactive particles in PAW on the experimental conditions of its preparation, namely the type of plasma source and its parameters and the composition of the working gas.

The effects of PAW on the fertigation of plants (i.e., the injection of fertilizers and chemicals into an irrigation system) are mentioned in review [46]. The exposure of seeds to hydrogen peroxide (H_2_O_2_) and nitric oxides (NO_x_) may trigger changes in plant hormones, promoting germination. Thus, PAW offers a unique opportunity to tailor and control the reactive species applied.

#### 1.3.1. Acidity and Hydrogen Peroxide

Acidity, caused by the formation of nitric acid (HNO_3_), and hydrogen peroxide are probably the only unquestionable and stable components of PAW.

#### 1.3.2. Ozone

The importance of ozone, undoubtedly a powerful disinfectant, in PAW is unclear, with studies providing differing reports as to its presence and concentration. It is obvious that its production depends on the experimental arrangement. In addition, this relatively labile particle decomposes or volatilizes easily and rapidly under certain conditions. This was confirmed, e.g., by [47]. On the other hand, Pavlovich et al. [48] found that the inactivation of *E. coli* correlated well with the ozone concentration in the aqueous phase, suggesting that ozone was the dominant species for bacterial inactivation under the conditions used. They found that for the low-power mode, the liquid chemistry was dominated by ozone, whereas for the high-power mode, hydrogen peroxide and nitric oxide species dominated.

According to Tarabová et al. [49], ozone is one of the abundant reactive species formed in air or oxygen plasma discharges, but it was not detected in the gas phase due to its thermal decomposition during the spark phase. Air transient spark (TS) discharge produces no aqueous ozone. Machala et al. [50] reported much higher O_3_ production rate in the streamer corona discharge than in the transient spark discharge, when it operates in the low-power ozone mode. A promising plasma source, producing large amounts of reactive oxygen species, including ozone, is the hydrodynamic cavitation plasma jet (HCPJ ozonizer) [51].

#### 1.3.3. Nitrous Acid

The kinetics of the reversible aqueous-phase decomposition of nitrous acid in water to NO and NO_2_ were described in detail in [52]. Studying the production of ROS and RNS in PAW, Tachibana and Nakamura [53] found that when O_2_ was incorporated into the reaction system, the main products became NO_2_^−^ and NO_3_^−^, while small amounts of H_2_O_2_ and NH_4_^+^ were detected only under low-O_2_ conditions and with water vapor. Of the main products, the predominance of NO_2_^−^ or NO_3_^−^ depends on the extent of the successive oxidation reactions from NO to NO_2_ and NO_3_ and on the competition between the gas-phase reaction rate and the rate of dissolution in water. Raud et al. [54] found that only negligible amounts of NO_2_ were generated during short (<5 min) plasma treatments in ambient air and a N_2_ atmosphere. The amount of nitrous acid (HNO_2_) in PAW can be significantly affected by its consumption in the reaction with hydrogen peroxide to form peroxonitrous acid, among other things. This process was described in detail in [55]. The interaction between nitric oxide and ozone is significant in dry gas but becomes less important in humid conditions.

#### 1.3.4. Peroxynitrous Acid (ONOOH)

For the general properties of this species, see [56]. Naïtali et al. [57] suggested the importance of peroxynitrous acid in an early paper, but they inferred its possible presence only from a slight decrease in the efficiency of artificial ROS and RNS mixtures that did not contain it. The detection of ONOO^−^/ONOOH is difficult because of their possible cross-reactivities and short lifetimes (they have a half-life of under 1 s) [58]. Kawasaki et al. [59] do not list peroxonitrite among the active ROS in their study. Machala et al. [50] described in detail the transport of primary gaseous RONS across the plasma–liquid interface and the formation of secondary RONS in water, namely peroxynitrite/peroxynitrous acid (ONOO^−^/ONOOH) and peroxynitrates/peroxynitric acid (O_2_NOO^−^/O_2_NOOH), and advocated their effectiveness in the resulting PAW. Julák [25] verified spectrophotometrically that peroxonitrite decomposes almost immediately in the acidic environment of PAW. Kutasi et al. [60,61] demonstrated that Cu ions originating from copper surfaces control the composition and ageing dynamics of plasma-activated liquids through a Fenton-type reaction. Peroxynitrous acid is formed and rapidly decays due to its short lifetime under acidic conditions, but it can be stabilized in a basic medium.

From the above selection of literature, it appears that the action of low-temperature plasma mostly produces PAW with an approximately similar qualitative composition and effect. However, when quantitative comparisons are made, it is evident that these parameters can vary significantly according to the preparation method, including the discharge and surrounding atmosphere. Hence, it can be concluded that the selected standard conditions must be replicated exactly when making detailed comparisons and/or searching for optimal conditions.

This paper summarizes an experiment using PAW as a chemical–physical stimulant for seed germination and early growth and as a disinfectant for the microbial decontamination of the wheat grain surface. Wheat grain was chosen as the test crop because it is a well-established experimental subject in connection with NTP. Due to this, we could effectively compare the obtained results with literary sources and draw the right conclusions. In this study, we attempted to mimic plasma-activated water by artificially mixing its known components to determine the effects of such mixtures on wheat seeds.

## 2. Results

The analysis of the plasma PAW provided the following results: The mean H_2_O_2_ content was 97.4 ± 3.2 mg/L, and the mean content of NO_3_^−^ was 391.1 ± 9.3 mg/L. The ozone (O_3_) content was below the lower limit of detectability of <3.8 mg/L. Only negligible amounts of nitrous acid (<10^−3^ mg/L of NO_2_^−^) were found. Semiquantitative estimation using indicator papers confirmed approximate values of 100 mg/L H_2_O_2_ and 250–500 mg/L NO_3_^−^ for plasma PAW and artificial PAW. The artificial PAW was prepared by dissolving H_2_O_2_ and HNO_3_ in the abovementioned concentrations in distilled water.

The measurements for all the characteristics regarding the germination and early growth of the wheat grains are summarized in Table 1. Significant increases in the treated samples were found for the following four characteristics: germination, length of shoot, and weight of fresh and dried shoot. The highest germination percentage (99.33%) and dried shoot weight (67.80 mg) were measured in the grains treated with PAW, representing approximately 107% and 138%, respectively, compared to the controls (100%). The highest shoot length (19.03 mm) and fresh shoot weight (498.20 mg) were obtained after artificial PAW treatment, which corresponded to approximately 120% and 131%, respectively, compared to the controls (100%).

On the other hand, significant decreases were found in the treated samples regarding the characteristics of root length, R/S length, R/S fresh weight, and R/S dry weight, for which the control samples always had the highest values. For the fresh and dried root weight, no statistically significant differences were recorded.

**Figure 1:**
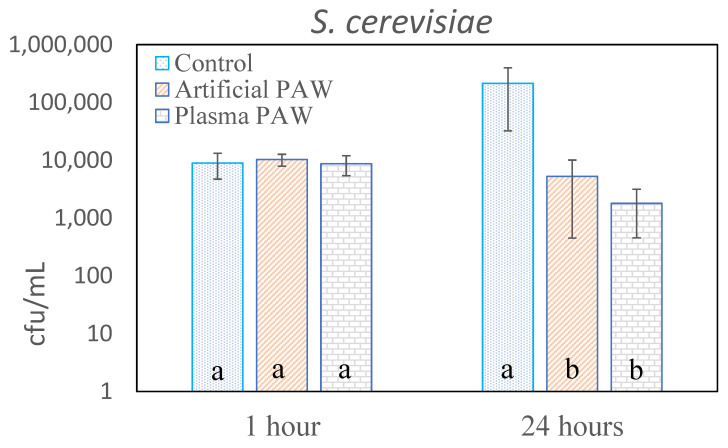
Inactivation of S. cerevisiae on inoculated wheat grains. Vertical bars indicate standard errors calculated from five parallel determinations. Significant differences at *p* < 0.05 are indicated by different letters. Separately determined results of PAW treatment after 1 h and 24 h are shown.

**Figure 2:**
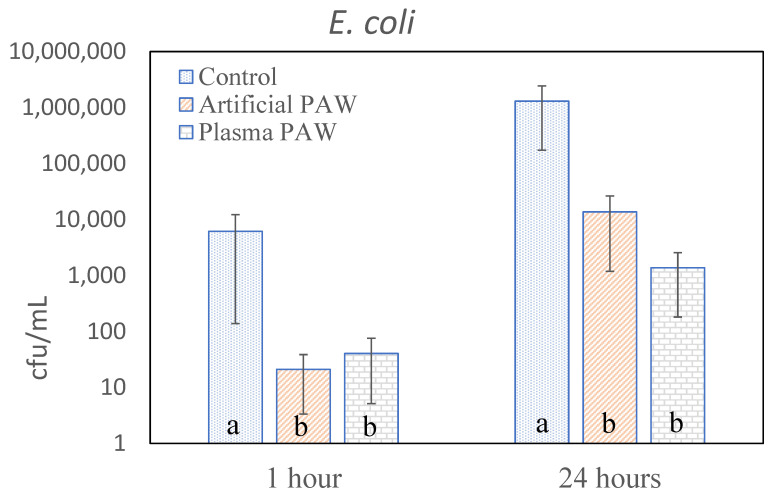
Inactivation of E. coli on inoculated wheat grains. Vertical bars indicate standard errors calculated from five parallel determinations. Significant differences at *p* < 0.05 are indicated by different letters. Separately determined results of PAW treatment after 1 h and 24 h are shown.

The results of the decontamination of the artificially contaminated grains are summarized in Figure 1 and Figure 2. In the case of Saccharomyces cerevisiae (Figure 1), the number of CFUs was not affected by exposure to PAW for 1 h, while exposure to both plasma PAW and artificial PAW for 24 h significantly reduced this number. In the case of Escherichia coli, a significant decontamination effect was already evident after exposure for 1 h; the somewhat less-pronounced effect after 24 h could be attributed to the high degree of contamination of the initial sample. In both cases, the initial CFU concentrations in the control samples were somewhat lower than the starting concentrations of 1.7·10^7^ or 5·10^6^ CFU/mL, respectively. This may have been due to the poor reproducibility of microbial extraction into SDW, probably because of the variable adhesion to the grain surface. Nevertheless, the effects of plasma PAW and artificial PAW were comparable within individual experiments; the small differences in their efficacy were not statistically significant in the cases of *S. cerevisiae* or *E. coli*.

## 3. Discussion

The concentrations of PAW active components measured here correspond well with those reported in our previous articles [25,30]. Despite the different analytical methods, the H_2_O_2_ content found here (97.4 ± 3.2 mg/L) is in good agreement with the previous value (126 mg/L), as is the content of NO_3_^−^ (391.1 ± 9.3 mg/L here vs. 327.6 mg/L previously). This also applies to the negligible amounts of O_3_ and nitrous acid (<10^−3^ mg/L and <0.1 mg/L, respectively). We did not previously assess the ozone (O_3_) content; here, its content was below the lower limit of quantification of <3.8 mg/L. However, this value is more of an estimate, due to the dubious reliability of the method used, which was mentioned in the Introduction. Regarding the lifetime of the PAW, in our earlier study [25], we found that the efficacy of PAW prepared by plasma treatment declined slightly after one year, probably due to the decay of the hydrogen peroxide. We expect the stability of artificial PAW to be comparable.

Concerning the disinfection of microbial contaminants, the difference between the effectiveness of PAW against bacteria and against yeast was conspicuous. While *E. coli* was inactivated after only one hour of exposure to PAW, this effect was only evident in *S. cerevisiae* after 24 h of exposure. However, this fact only confirms previously reported findings regarding the varying sensitivity of microorganisms, namely bacteria and fungi, to plasma. An important finding was the equal sensitivity of individual organisms to PAW prepared by plasma treatment and artificially prepared PAW. Although the sensitivity of *E. coli* to plasma PAW was slightly higher, this difference was not statistically significant.

The significantly higher number of germinating wheat grains in the PAW pre-treated samples corresponds to the results of other works [62,63,64,65,66]. It has been shown in many experiments that shorter durations of direct NTP treatment can positively affect the germination of different plant species [67,68,69,70,71,72]. PAW treatment can even improve seed germination and plant growth under stress conditions [73].

The allocation of biomass to roots and stems is one of the most frequently measured growth characteristics; therefore, different R/S (root/shoot) ratios are monitored variables in plant production biology. In agricultural practice, the R/S ratio is related to the genetically fixed properties of cultivated plants, such as the accumulation of nutrients in the targeted organs (seeds, tubers, etc.); the resistance to drought; and the ability to use water efficiently. In terms of the efficiency of the use of environmental resources in the production process, the optimization of this R/S ratio is crucial, but still relative [74,75]. The results of this study showed that wheat grains after PAW treatment invested in their aboveground parts rather than in their roots. The parameters R/S length, R/S fresh weight, and R/S dry weight were significantly lower in the treated samples than in the control samples (Table 1). Although the plants had significantly longer and heavier (fresh) parts compared to the control samples, this cannot be seen as a positive effect of the treatment on plant development. These investments in the aboveground parts indicate a stress-induced adverse reaction of the germinating plant. In this experiment, PAW should be considered a stress factor that caused unwanted responses [76].

## 4. Materials and Methods

### 4.1. PAW Preparation

**Figure 3:**
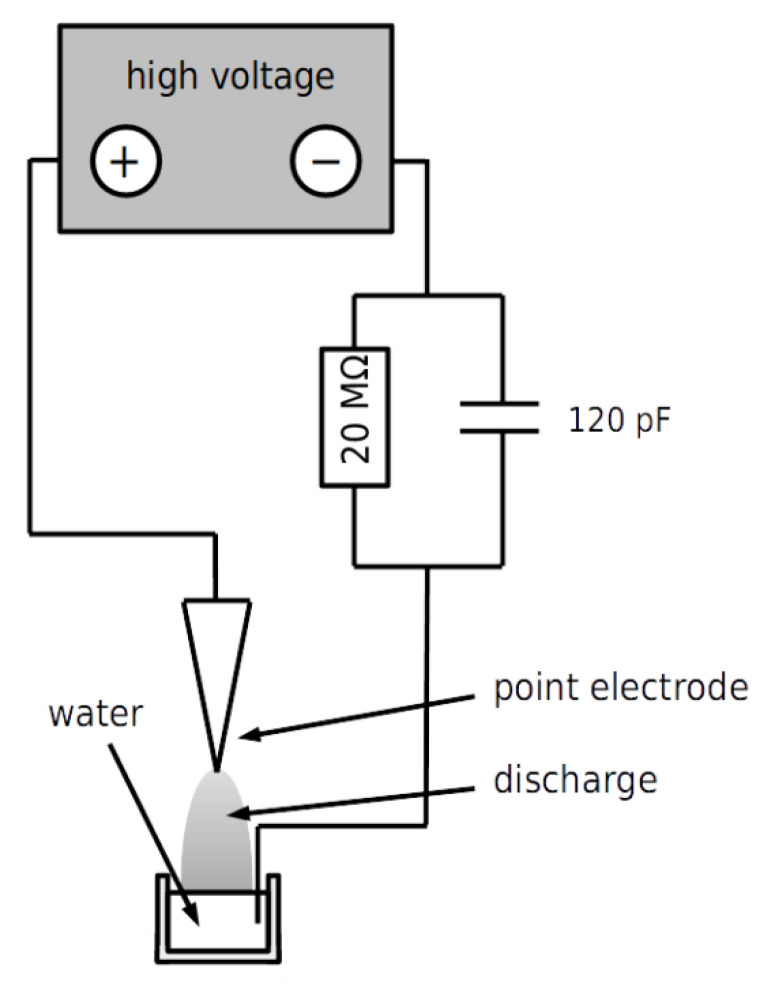
Schematic drawing of the PAW-generating apparatus.

PAW (denoted here as plasma PAW) was prepared by plasma exposure and analyzed in the same manner as previously described by [24,25,30]. One mL of demineralized water was exposed using point-to-plane DC corona discharge stabilized by a 20 MΩ ballast resistor and a 25 pF capacitor in a regime of positive transient spark at 9 kV for 30 min. The ground electrode comprised the surface of the water grounded with an immersed platinum wire. The distance between the tip of the needle and the plate electrode was 3 mm, adjusted by a micrometric screw to obtain an average current of 300 µA. The experimental arrangement is depicted schematically in Figure 3 and described in detail in [77]. To produce a sufficient amount of PAW, the exposure of 1 mL of water was repeated and resulting batches were pooled.

### 4.2. PAW Analysis

The method for determining the PAW composition was identical to that used in [30]: the acidity and the contents of NO_3_^−^ (or HNO_3_), NO_2_^−^ (or HNO_2_), and H_2_O_2_ were measured. Briefly, pH was determined by alkalimetric titration with 0.001 M NaOH solution, and the equivalency was indicated by pH measurement using a glass electrode.

The content of NO_2_^−^ (or HNO_2_) was determined by the Griess reaction, i.e., diazotization with sulfanilic acid (SA) and copulation with N-(1-naftyl)-ethylendiamindihydrochlorid (NED). After adding NED to PAW, transmittance at 550 nm was measured spectrophotometrically.

The content of NO_3_^−^ (or HNO_3_) was obtained by subtracting the content determined by the Griess reaction from the total acidity determined by alkalimetric titration. For the semiquantitative estimation of NO_2_^−^ and NO_3_^−^, test strips (Quantofix 913 13, Macherey-Nagel, Duren, Germany) for nitrate (10–500 mg/L) and nitrite (1–80 mg/L) were used.

The content of H_2_O_2_ was determined by oxidizing titanium sulphate (Ti(SO_4_)_2_) to yellow pertitatic acid (H_2_TiO_4_). After adding the Ti(SO_4_)_2_ reagent to the PAW, transmittance was measured at 410 nm. The presence of H_2_O_2_ was tested semiquantitatively using Quantofix Peroxide 25 and 100 test strips (Macherey-Nagel, Duren, Germany), based on peroxidase enzymatic activity.

For ozone (O_3_) measurement, iodometric titration was chosen: 0.02 M standard KI solution and starch solution were added to PAW acidified with H_2_SO_4_. The resulting iodine (I_2_) was retitrated with 0.001 M thiosulphate (Na_2_S_2_O_3_) solution. Because the same reaction takes place with hydrogen peroxide, the concentration of O_3_ was obtained by subtracting the H_2_O_2_ concentration determined from the abovementioned H_2_TiO_4_ photometry.

### 4.3. Artificial PAW Preparation

The artificial PAW was prepared by adding 323 mg of 30% hydrogen peroxide solution and 602 mg of 65% nitric acid solution to 1 L of distilled water. These quantities correspond to the concentrations found in Section 2 (Results), i.e., 97.4 ± 3.2 mg/L of H_2_O_2_ and 391.1 ± 9.3 mg/L of NO_3_^−^; the negligible amount of NO_2_^−^ (<10^−3^ mg/L) was omitted.

### 4.4. Germination and Early Growth Tests

Grains of Frisky var. winter wheat (*Triticum aestivum* L.) were obtained from Osiva Boršov, with 93% germination and a weight of 50 g per thousand grains. Only visually healthy and undamaged grains without husks were used.

The germination and early growth tests were performed on three layers of filter paper in a 9 cm diameter petri dish. Thirty seeds per dish were placed on filter paper moistened with 3 mL of water, freshly prepared PAW, and artificial PAW, respectively. One mL of water/PAW/artificial PAW was used every day for irrigation, with a cultivation regime in darkness at 23 °C. Thirty seeds irrigated with distilled water served as the control. All experiments were repeated five times in parallel. Each test and control variant contained 150 wheat grains.

The number of germinating seeds was counted every day, and the shoot length, root length, and fresh weights were measured on the fifth day of cultivation. The shoots and roots were then dried separately at 60 °C for 24 h and the weights of the dried shoots and roots were measured.

From the values of the characteristics obtained on the fifth day of cultivation, the ratios between the underground (R) and aboveground (S) parts of the wheat seedlings were determined. The lengths of the measured parts, their fresh weight, and their dry weight were included in the R/S ratio. Thus, the following characteristics were obtained: R/S length, R/S fresh weight, and R/S dry weight. All characteristics of R/S ratios were calculated according to [78].

### 4.5. Inoculum Preparation

Strains of *Escherichia coli* and *Saccharomyces cerevisiae* were used as representatives of bacteria or yeast, respectively. They were incubated on Mueller–Hinton (MH) nutrient agar (Oxoid, Brno, Czech Republic) for 48 h at 37 °C (*E. coli*) and on Sabouraud (S) dextrose agar (Oxoid, Brno, Czech Republic) for 4 days at 25 °C (*S. cerevisiae*). A loopful of biomass was harvested and suspended in sterile distilled water, and the concentration of *E. coli* was adjusted to approximately 1.7 × 10^7^ colony-forming units (CFU) per mL; the yeast concentration was 5 × 10^6^ CFU/mL.

### 4.6. Grain Inoculation

Before inoculation, the grains were decontaminated by immersion in a 500 mg/L sodium hypochlorite solution for 30 s. The inoculation was then carried out by immersing 8 mg of grains in 20 mL of bacterial or yeast suspension, respectively, for 30 min. The bacterial suspension was then drained, and the grains were distributed in a single layer on sterile filter paper and allowed to dry for 90 min in a laminar flow cabinet at 25 °C.

### 4.7. Grain Treatments

Portions of 0.25 g of inoculated grains were submerged in parallel in 1 mL of plasma PAW or 1 mL of artificial PAW for 1 h or 24 h, respectively. Another 0.25 g of grains was submerged in 1 mL of sterile distilled water (SDW) as an untreated control. All treatments were performed five times. Immediately after each treatment, the liquids were drained, and the grains were transferred into 1 mL of pure SDW. These mixtures were vortexed for 30 s, and 0.6 mL of this SWD containing the extracted microbes was inoculated on Sabouraud agar (*S. cerevisiae*) or Mueller–Hinton agar (*E. coli*). After incubation, the number of colony-forming units was counted.

### 4.8. Data Analysis

All measurements were repeated five times, and the obtained data were analyzed using STATISTICA software (Statistica 13, StatSoft Inc., Tulsa, OK, USA) at a significance level of 0.05. Logarithmic transformation (y = log(x)) of the basic data was used for normalization prior to analysis.

One-way analysis of variance (ANOVA) was used to evaluate the influence of the treatments on the characteristics of seed germination and early growth. The detailed testing of experimental variances was carried out using Tukey’s honest significant difference (HSD) test. The data from the Tukey test are presented in detail.

## 5. Conclusions

The results showed improvements in some seed parameters, achieved not only by the direct exposure of seeds to plasma but also by indirect exposure to PAW. These changes were probably due to the decontamination of the grains. The application of this finding in agriculture could be practically significant. A comparison of the properties of PAW-exposed and unexposed grains showed that PAW exposure somewhat increased germination, shoot length, and fresh and dry shoot weight. Root length and R/S length somewhat decreased, while the other parameters changed only irregularly or not at all. Regarding the decontamination of artificially contaminated grains, the results of the plasma PAW and artificial PAW were comparable; the higher efficacy of plasma PAW against *S. cerevisiae* and artificial PAW against *E. coli* was not statistically significant. A review of the literature showed that the composition of PAW depends strongly on the experimental conditions of its preparation, in particular the type of production discharge and the composition of the atmosphere. The results reported here confirm that there is no significant difference between the effects of PAW prepared by exposure to plasma and those of PAW prepared by the mixture of its stable components.

## Figures and Tables

**Table 1:** Characteristics of seed germination and early growth of wheat grains after three types of treatment: water, artificial PAW, and plasma PAW. The mean, standard error (SE), and Tukey’s test results are given. Significant differences at *p* < 0.05 are indicated by different letters.

Treatment	Germination (%)	Length of Root (mm)	Length of Shoot (mm)	Weight of Fresh Root (mg)	Weight of Fresh Shoot (mg)
	Mean ± SE	Mean ± SE	Mean ± SE	Mean ± SE	Mean ± SE
Control	92.67 ± 1.88 a	36.63 ± 1.05 a	15.97 ± 0.42 a	578.80 ± 26.66 a	380.20 ± 15.25 a
Artificial PAW	94.00 ± 1.85 ab	26.38 ± 1.17 b	19.03 ± 0.59 b	575.20 ± 31.95 a	498.20 ± 26.80 b
Plasma PAW	99.33 ± 1.75 b	23.06 ± 1.00 b	18.42 ± 0.53 b	491.00 ± 34.29 a	497.00 ± 16.65 b
**Treatment**	**Weight of Dried Root (mg)**	**Weight of Dried Shoot (mg)**	**R/S Length**	**R/S Fresh Weight**	**R/S Dry Weight**
	**Mean ± SE**	**Mean ± SE**	**Mean ± SE**	**Mean ± SE**	**Mean ± SE**
Control	64.40 ± 2.42 a	49.00 ± 1.58 a	2.30 ± 0.04 a	1.53 ± 0.06 a	1.23 ± 0.06 a
Artificial PAW	67.00 ± 1.97 a	62.20 ± 2.40 b	1.31 ± 0.08 b	1.15 ± 0.01 b	1.08 ± 0.02 ab
Plasma PAW	66.20 ± 2.18 a	67.80 ± 2.18 b	1.25 ± 0.03 b	0.98 ± 0.05 c	0.98 ± 0.03 b

## Data Availability

Not applicable.
